# Three-Dimensional Surgical Strategy Planning With 3D Slicer for Redo Coronary Artery Bypass Grafting After Acute Aortic Dissection Repair

**DOI:** 10.1016/j.atssr.2026.03.012

**Published:** 2026-04-01

**Authors:** Shun-Ichiro Sakamoto, Yoshiyuki Watanabe, Tomohiro Murata, Motohiro Maeda, Yosuke Ishii

**Affiliations:** 1 Department of Cardiovascular Surgery, Nippon Medical School Musashikosugi Hospital, Kanagawa, Japan; 2 Department of Cardiovascular Surgery, Nippon Medical School, Tokyo, Japan

## Abstract

Redo coronary artery bypass grafting after thoracic aortic surgery is challenging because of altered anatomy and dense adhesions. We report redo revascularization for right coronary ischemia 3 months after repair of acute type A aortic dissection. Contrast-enhanced computed tomography analyzed with 3D Slicer localized the right coronary artery crux relative to the thoracic cage and simulated right gastroepiploic artery routing and length. This enabled a sternotomy-sparing, off-pump right gastroepiploic artery to right coronary artery bypass with uneventful recovery; postoperative computed tomography confirmed graft patency.

Three-dimensional (3D) image processing software, including 3D Slicer, is increasingly used for patient-specific preoperative planning in cardiovascular surgery.^1^ In redo coronary surgery, precise anatomic understanding is critical for selecting an appropriate conduit, identifying a safe target, and planning access in the setting of adhesions and limited exposure.^2^

A 52-year-old man underwent ascending aortic replacement for Stanford type A acute aortic dissection with concomitant right axillary artery bypass and coronary artery bypass grafting (aorta–saphenous vein graft to the right coronary artery [RCA] segment 2). Three months later, he presented with chest tightness and inferior ST-segment elevation. Contrast-enhanced computed tomography showed occlusion of the saphenous vein graft and perigraft fluid around the ascending aortic prosthesis. The RCA opacified distally but was narrowed from the ostium to segment 2, and percutaneous intervention was not feasible. Given suspected severe mediastinal adhesions and the retrosternal course of the prosthetic axillary bypass graft, redo coronary bypass was planned by a sternotomy-sparing approach.

With use of 3D Slicer (version 5.8.1), a 3D reconstruction of the thoracic cage, heart, coronary arteries, liver, and right gastroepiploic artery (RGEA) was created from contrast-enhanced computed tomography (Figure). The RCA crux was localized relative to the ribs, diaphragm, and chest wall, and the available RGEA length was measured by centerline analysis. On the contrast-enhanced model, the luminal diameter of the RGEA was approximately 2 mm, reflecting the opacified lumen rather than the external vessel diameter. The reconstruction indicated that at least 20 cm of conduit could be harvested, and the simulated graft route from the upper abdomen to the RCA crux measured approximately 16 cm, confirming sufficient length for revascularization and guiding the optimal incision line from the right fourth intercostal space to the upper abdomen (reversed-J configuration). For illustrative purposes, the thoracic cage model was additionally postprocessed by digital deformation in Adobe Photoshop (Adobe Inc) to approximate rib spreading after right-sided thoracotomy. A Video demonstrating the 3D planning process is provided. Intraoperatively, although the vessel diameter was not formally measured, a suitable segment was harvested, yielding a 25-cm graft that was judged adequate for anastomosis on the basis of direct surgical inspection.

**Figure:**
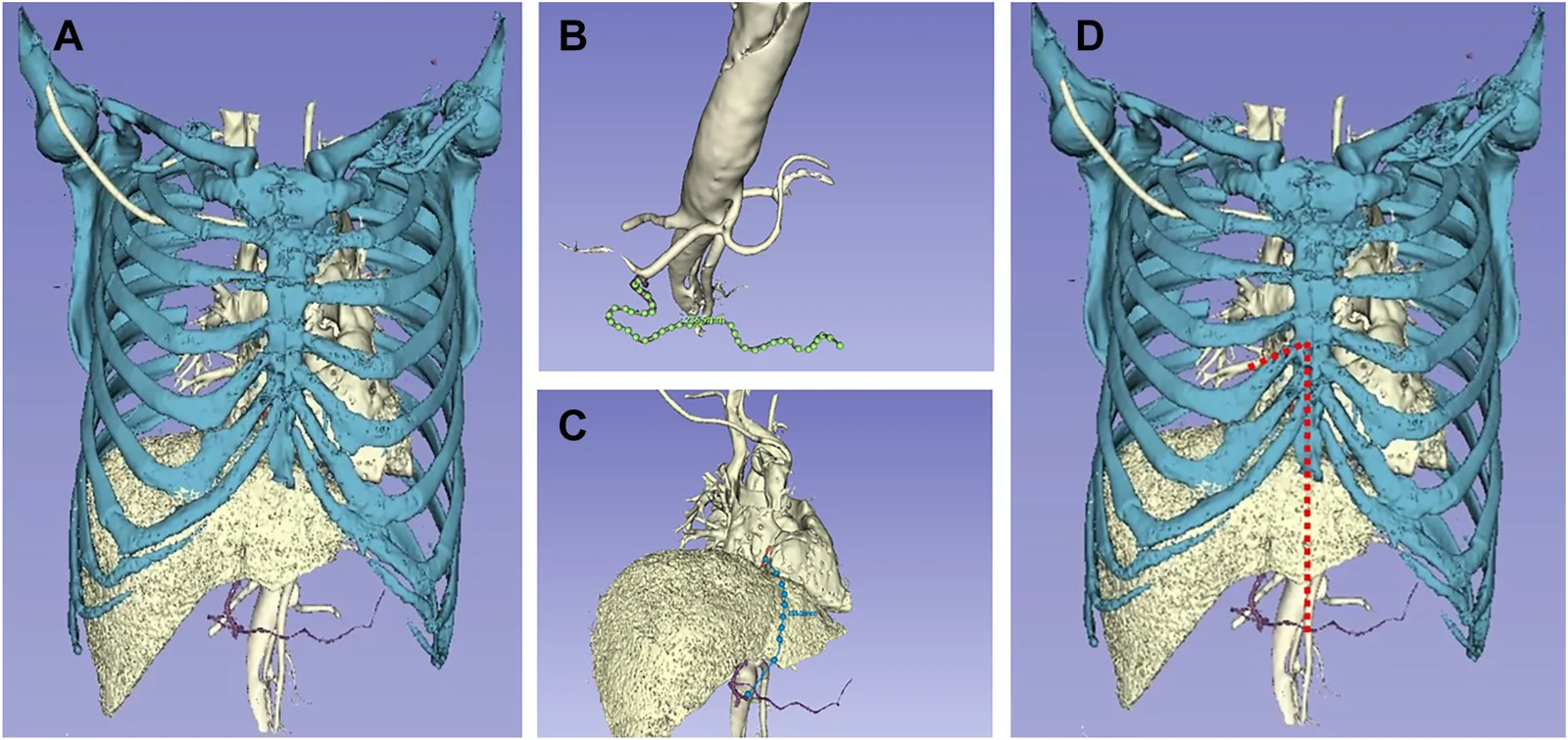
Three-dimensional (3D) surgical strategy planning with 3D Slicer. (A) Composite 3D reconstruction showing the thoracic cage, heart and great vessels, right coronary artery target (red), liver, and right gastroepiploic artery (RGEA; purple), with the retrosternal axillary bypass graft. (B) RGEA length measured by centerline analysis (green). (C) Simulated RGEA routing to the right coronary artery crux (segment 3) with the anticipated graft trajectory (blue) and target site (red). (D) Planned access line based on 3D simulation (red dashed line).

Surgery was performed through the planned incision. After laparotomy, the RGEA was harvested and mobilized for 25 cm from the branch point of the superior pancreaticoduodenal artery. Dense epicardial adhesions were encountered; the prior vein-graft anastomosis was identified and used as a landmark to dissect the RCA distally and to expose the target segment (segment 3/crux). Under stabilization, an RCA occlusion test caused no arrhythmia or hemodynamic compromise, and an off-pump RGEA-RCA anastomosis was completed with 7-0 polypropylene. After reperfusion, proximal bleeding was controlled with a vascular clip. Transit-time flow measurement confirmed satisfactory graft flow.

The patient was extubated on the day of surgery and had an uneventful recovery. He was discharged home on postoperative day 11. Follow-up coronary computed tomography demonstrated a patent RGEA graft.

## Comment

Redo coronary artery bypass grafting remains technically challenging because of distorted anatomy, dense adhesions, and limited exposure, particularly for the deeply located RCA.^2^ Intrapericardial adhesions evolve through fibrin deposition, fibroblast activation, collagen deposition, and neovascularization, which can result in firm scarring within weeks after surgery.^3^

In this patient, redo surgery was required only 3 months after type A dissection repair, when adhesions can already be mature. Perigraft fluid around the ascending aortic prosthesis suggested ongoing postoperative inflammatory change and increased concern for difficult dissection while necessitating careful exclusion of graft infection or sterile perigraft seroma.^4^^,^^5^

Several factors increased the risk of conventional reentry and favored alternative planning: prior complex aortic repair, a retrosternal prosthetic axillary bypass, early reoperation, and the need for a nonstandard conduit (RGEA).^6^^,^^7^ In this setting, 3D Slicer–based strategy planning allowed integration of conduit selection, target localization (RCA crux), simulated graft routing with length assessment, and incision design based on the spatial relationship between the target and the thoracic cage.

Three-dimensional, patient-specific modeling has been increasingly used in cardiac surgery to facilitate procedure planning in anatomically complex cases. In particular, software-based platforms built on 3D Slicer have enabled virtual planning and simulation workflows for intracardiac operations, demonstrating their utility in translating cross-sectional imaging into actionable surgical strategy.^8^ Whereas 3D printing can be useful for visualization, software-based 3D planning allows rapid iteration without fabrication time and can be readily adapted to patient-specific anatomy.^1^^,^^8^ Although our reconstructions were based on static computed tomography and required manual centerline definition, the approach may be useful for other high-risk redo operations in which standard access is hazardous.

In conclusion, 3D surgical strategy planning using 3D Slicer enabled safe, sternotomy-sparing redo RCA bypass with an RGEA conduit after acute aortic dissection repair.
